# Ubiquitin Fusion Degradation Protein 1 as a Blood Marker for The Early Diagnosis of Ischemic Stroke

**Published:** 2007-04-19

**Authors:** Laure Allard, Natacha Turck, Pierre R. Burkhard, Nadia Walter, Anna Rosell, Marianne Gex-Fabry, Denis F. Hochstrasser, Joan Montaner, Jean-Charles Sanchez

**Affiliations:** 1 Biomedical Proteomics Research Group, Department of Structural Biology and Bioinformatics, Medical University Center, CH-1211 Geneva 4, Switzerland; 2 Neurology Department, Geneva University Hospital, CH-1211 Geneva 14, Switzerland; 3 Biomedical Proteomics Research Group, Central Clinical Chemistry Laboratory, Geneva University Hospital, CH-1211 Geneva 14, Switzerland; 4 Neurovascular Research Laboratory, Stroke Unit, Vall d’Hebron Hospital, Barcelona, Spain; 5 Clinical Research Unit, Department of Psychiatry, CH-1225 Chêne-Bourg, Switzerland; 6 Pharmacy Section, Faculty of Sciences, Geneva University

**Keywords:** Diagnosis, Plasma Markers, Stroke, UFD1, Brain Damage

## Abstract

**Background:**

Efficacy of thrombolysis in acute ischemic stroke is strongly related to physician’s ability to make an accurate diagnosis and to intervene within 3–6 h after event onset. In this context, the discovery and validation of very early blood markers have recently become an urgent, yet unmet, goal of stroke research. Ubiquitin fusion degradation protein 1 is increased in human postmortem CSF, a model of global brain insult, suggesting that its measurement in blood may prove useful as a biomarker of stroke.

**Methods:**

Enzyme-linked immunosorbent assay (ELISA) was used to measure UFD1 in plasma and sera in three independent cohorts, European (Swiss and Spanish) and North-American retrospective analysis encompassing a total of 123 consecutive stroke and 90 control subjects.

**Results:**

Highly significant increase of ubiquitin fusion degradation protein 1 (UFD1) was found in Swiss stroke patients with 71% sensitivity (95% CI, 52–85.8%), and 90% specificity (95% CI, 74.2–98%) (*N* = 31, *p* < 0.0001). Significantly elevated concentration of this marker was then validated in Spanish (*N* = 39, *p* < 0.0001, 95% sensitivity (95% CI, 82.7–99.4%)), 76% specificity (95% CI, 56.5–89.7%)) and North-American stroke patients (*N* = 53, 62% sensitivity (95% CI, 47.9–75.2%), 90% specificity (95% CI, 73.5–97.9%), *p* < 0.0001). Its concentration was increased within 3 h of stroke onset, on both the Swiss (*p* < 0.0001) and Spanish (*p* = 0.0004) cohorts.

**Conclusions:**

UFD1 emerges as a reliable plasma biomarker for the early diagnosis of stroke, and in the future, might be used in conjunction with clinical assessments, neuroimaging and other blood markers.

## Introduction

While stroke remains a leading cause of death and disability in industrialized countries, it has been demonstrated that a rapid assessment and early therapeutic intervention may improve the management and the long-term prognosis of stroke patients (Khaja and Grotta, 2007). At present, a diagnosis of stroke relies on physician’s neurological examination and neuroimaging techniques such as brain CT scan and/or MRI (Chalela et al. 2007). CT scan succeeds at detecting hemorrhagic stroke but usually fails at demonstrating ischemic lesions in their early phase. MRI can detect aspects of stroke within a few hours, but such sophisticated and expensive tools are available only in specialized hospitals. Rapid diagnosis and management of stroke remains a challenge as it suffers from a lack of reliable biological markers. An early diagnostic blood marker of stroke is expected to allow more rapid and appropriate therapeutic interventions and may possibly reduce the extent of tissue damage, disability and risk of death. Indeed, an efficient thrombolytic treatment can only be considered within the crucial first 3–6 h of the onset of symptoms (Albers and Olivot, 2007). Over the past few years, a number of blood markers including tau (Zemlan et al. 1999; Hesse et al. 2001; Bitsch et al. 2002), neuron specific enolase (NSE), B-type neurotrophic growth factor (B-NGF) (Persson et al. 1987; Cunningham et al. 1991; Zimmermann-Ivol et al. 2004), astroglial protein S-100b (Persson et al. 1987; Reynolds et al. 2003; Lynch et al. 2004), glial fibrillary acidic protein (GFAP) (Herrmann et al. 2000), C-reactive protein (CRP) (Rost et al. 2001; Silvestri et al. 2004; Di Napoli et al. 2001; Intiso et al. 2004; Smith et al. 2004; Pedersen et al. 2004), matrix metalloproteinase (MMP9) (Lynch et al. 2004; Montaner et al. 2001; Reynolds et al. 2003), vascular and intracellular cell adhesion molecules (VCAM or ICAM) (Lynch et al. 2004; Pedersen et al. 2004), tumor necrosis factor-α (TNF-α) and interleukins (IL-1, IL-6 and IL-8) (Pedersen et al. 2004; Intiso et al. 2004; Silvestri et al. 2004; Smith et al. 2004) have raised interest in the diagnosis and prognosis of stroke. Nevertheless, limitations for these markers are that (i) they displayed either low sensitivity or specificity in differentiating stroke and control patients, (ii) they were tested in limited samples with similar genetic background, ranging from a few patients to 223 for the most extensive study by Reynolds et al. 2003 or (iii) they are released in blood relatively late after stroke onset.

The main objective of the present study was to evaluate ubiquitin fusion degradation protein 1 (UFD1) as a new blood marker that could help in the early diagnosis of stroke patients. We have recently explored postmortem cerebrospinal fluid (CSF) as a model of massive brain insult (Lescuyer et al. 2004). CSF is an ideal fluid to use in the search of biomarkers of brain damaged-associated disorders. Owing to the close proximity of CSF to the brain, changes that are detected in the protein composition of CSF often reflect changes that currently occurred or have previously occurred in the brain. For example, heart-fatty acid binding protein (H-FABP) was initially found over-expressed in postmortem compared to antemortem CSF and was validated as a potential biomarker of Creutzfeld–Jakob disease (Guillaume et al. 2003) and stroke (Zimmermann-Ivol et al. 2004). More recently, RNA binding protein regulatory subunit (PARK7) and nucleotide diphosphate kinase A (NDKA), also identified in postmortem CSF were validated as early plasmatic markers of stroke (Allard et al. 2005). UFD1 was similarly found over-expressed in postmortem compared to ante-mortem CSF. Altogether, the above-mentioned data suggested that UFD1 could also be released in the blood of patients affected by a brain injury. Here, we report the evaluation of UFD1 levels in three independent cohorts, demonstrating its utility as a blood biomarker of stroke.

## Materials and Methods

### Blood samples for UFD1 validation

Plasma and sera samples were obtained from two European (Swiss and Spanish) and one North-American cohort of patients, which were used for the assessment of UFD1 level.

The local institutional ethical committee board of each centre approved the clinical protocol. The diagnosis of stroke was established by a trained neurologist and was based on the sudden appearance of a focal neurological deficit and the subsequent delineation of a lesion consistent with the symptoms on brain CT or MRI images, with the exception of transient ischemic attacks (TIAs) where a visible lesion was not required for diagnosis. After centrifugation at 1500 *g* for 15 min at 4 °C, heparin-plasma samples were aliquoted and stored at −80 °C until analysis. For the sera, blood samples were allowed to clot for 15 min at room temperature and were then centrifuged for 5 min at 1500 *g* at 4 °C. Samples were aliquoted and stored at −80 °C until analysis. Analyses were performed on frozen samples.

### Swiss cohort

Sixty-six consecutive Swiss stroke and control patients admitted to the Geneva University Hospital emergency unit were enrolled in this study between August 1996 and January 1997 (Table 1). Two stroke patients were excluded, as they finally did not have a stroke. Two control patients were also excluded from the study because of sustaining a stroke shortly after the blood withdrawal. Of the 62 consecutive patients enrolled, 31 were diagnosed with non-neurological conditions and classified as control samples (21 men and 10 women, average age of 70.26 years, range 28–91 years) and 31 were diagnosed with stroke (23 men and 8 women, average age of 71.81 years, range 25–92 years) including 28 ischemic (among them 6 TIAs, 22 established) and 3 intra-cerebral hemorrhagic strokes. The control group included patients with various medical or surgical conditions, including cancer (*n* = 12) and gastro-intestinal disorders. A few of them suffered from chronic neurological conditions as secondary diagnosis, including meningioma (*n* = 1) and dementia (*n* = 3). None of them had a past or recent history of cerebrovascular event. For each patient, a blood sample was collected at the time of admission in dry heparin-containing tubes. For the patients of the stroke group, the average time interval between the neurological event and the first blood draw was 20.11 h (range 30 min to 5 days).

### Spanish cohort

Twenty-nine control and 39 stroke patients were enrolled in this study (Table 1). Tests were performed on sera samples. The stroke subgroup included 10 hemorrhagic and 29 ischemic patients. The ischemic population was divided into (i) cardioembolic among them partial (*n* = 5) and total (*n* = 4) anterior circulation infarct, (ii) atherothrombotic among them partial (*n* = 5) and total (*n* = 5) anterior circulation infarct and (iii) lacunar infarct (*n* = 5) and TIA (*n* = 5). The 39 stroke patients were recruited within 24 h after onset of symptoms, and exact time was obtained for 18 patients. The average time interval between the neurological event and the first blood draw for these patients was 10.0 h (range 30 min to 6.25 days).

### North-american cohort

A North-American cohort was included and studied as described by Reynolds et al. Briefly, it was composed of 30 control and 53 stroke patients (including 6 hemorrhagic, 23 TIAs and 24 established ischemic strokes). Blood samples were collected within 24 h after the onset of symptoms (Table 1).

### Sandwich ELISA Immunoassay procedure

The ELISA was performed using 96-well Reacti-Bind^™^ NeutrAvidin^™^ coated Black Plates (Pierce, Rockford, IL). Plates were first rinsed in borate buffer saline pH 8.4 (BBS) (100 mM H_3_BO_3_, 25 mM Na_2_B_4_O_7_ (Sigma, St. Louis, MO, USA) 75 mM NaCl (Merck, Darmastadt, Germany)) on a NOVAPATH^™^ washer (Bio-Rad, Hercules, CA). Then, 50 μL of UFD1 antibody-biotin conjugated (2 μg/mL) (Biosite, California, USA) prepared in the dilution buffer A at pH 7 (DB, Polyvinyl Alcohol, 80% hydrolyzed, *M*_W_ 9000–10,000 (Aldrich, Milwaukee, WI, USA), MOPS (Sigma), NaCl, MgCl_2_ (Sigma), ZnCl_2_ (Aldrich), pH 6.90, BSA 30% Solution, Manufacturing Grade (Serological Proteins Inc., Kankakee, IL)), were added and incubated for 1 h at 37 °C. Plates were then washed three times in BBS in the plate washer. The recombinant protein UFD1 (Biosite, California, USA) was diluted at 100 ng/mL in the dilution buffer A. Plasma and sera samples were diluted twice in the dilution buffer A. Fifty microliter of antigen was then added and incubated for 1 h at 37 °C. After washing, 50 μL of alkaline phosphatase conjugated UFD1 antibodies (Biosite, California, USA) was added at the appropriate dilution in the dilution buffer A and incubated for 1 h at 37 °C. The 96-well plate was then washed three times with BBS in the plate washer and 50 μL of fluorescence Attophos^®^ AP Fluorescent substrate (Promega, Madison, WI) was added. Plates were read immediately on a SpectraMax GEMINI-XS (Molecular Devices Corporation, Sunnyvale, CA, USA) fluorometer microtiter plate reader using the endpoint mode relative fluorescence units (RFU) (λ_excitation_ = 444 nm and λ_emission_ = 555 nm). The European cohorts were tested in Geneva (CH) on 96-well microtiter plates and the North-American cohort in San Diego (CA) on a fully automated TECAN platform using 384-well microtiter plates. Each sample was assayed in duplicate and distributed randomly on the plate. The intra- and inter-run coefficient of variations was always under 10%. Calibration curves were performed in the same plate using the recombinant protein. The UFD1 recombinant protein was diluted to concentrations of 100, 50, 25, 12.5, 6.25, 3.125, 1.56 and 0 μg/L in the dilution buffer in Geneva and in control plasma in San Diego. A calibration curve was performed using a linear regression in the linear range of the curve (1.56–50 μg/L). Protein levels were initially expressed in relative fluorescence units (RFU) and the concentrations were calculated *via* the calibration curve.

### Statistical analysis

Statistical analyses were performed using GraphPad Prism^®^ software version 4.0 (GraphPad Software Inc., San Diego, C.A, U.S.A.) and graphs were produced with Aabel 1.5.8 software (Gigawiz Ltd. Co.). Notched box and whisker charts were drawn, that shows 10th, 25th, 50th, 75th and 90th percentiles and outliers. The diamond-shape indicates the mean. The notches in the boxes correspond to the median and its confidence interval. Boxes whose notches do not overlap indicate that the medians of the two groups differ at the 5% significance level. To assess the performance of protein levels to discriminate between stroke and control samples, a non-parametric Mann–Whitney test was performed. For comparing sub-samples according to the time interval between blood samples and onset of symptoms, a one-way Kruskal–Wallis ANOVA followed by Dunn’s multiple comparison tests was carried out. Receiver operating characteristic (ROC) curve analyses were performed and cut-off (CO) values were obtained from the curves. When possible, optimal threshold value was chosen at specificity ideally above 90%, as this parameter is the most clinically relevant to rule-in ischemic stroke patients. Odds ratios (OR) were also calculated for each sample. A Fisher’s exact test and a Mann–Whitney test were used to check, respectively, if sex and age did not differ significantly between stroke and controls. Comparison of ROC curves was performed using MedCalc version 8.2.

## Results

Ubiquitin fusion degradation protein 1 (UFD1, Swiss-Prot Accession No. Q92890 of 343 AA, 38725 Da and theoretical pI 5.96) was measured and validated in three independent cohorts.

**Figure 1:**
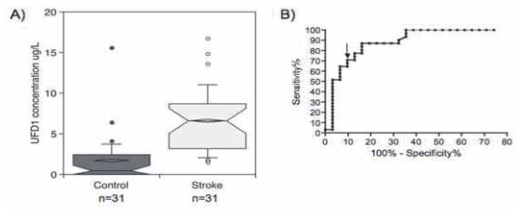
(A) UFD1 concentrations in stroke vs. control Swiss patients. Notched box and whisker charts show 10th, 25th, 50th, 75th and 90th percentiles and outliers. The diamond-shape indicates the mean. (B) UFD1 ROC curve. The arrow indicates the cut-off that gives the best sensitivity and specificity described in Table 2.

**Figure 2:**
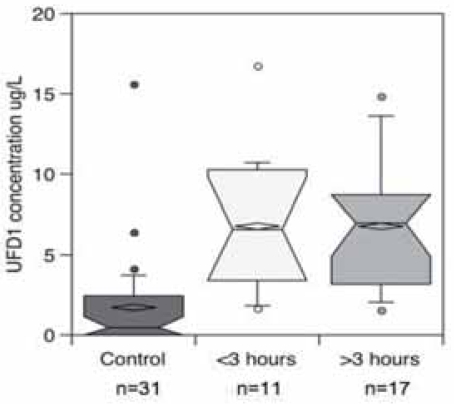
UFD1 concentrations as a function of time after the onset of the symptoms (Ischemic and TIA patients) for the Swiss population. Notched box and whisker charts show 10th, 25th, 50th, 75th and 90th percentiles and outliers. The diamond-shape indicates the mean.

A Swiss cohort comprising 62 consecutive patients and described in Table 1, was investigated. Stroke and control patients did not statistically differ with respect to age (*p* = 0.725, Mann–Whitney test) and sex (*p* = 0.78, Fisher’s exact test). UFD1 was evaluated for its ability to differentiate stroke and control patients, irrespective of stroke type, lesion size or location. Figure 1A shows a highly significant increase of UFD1 in the stroke samples compared to the controls (Mann–Whitney test, *p* < 0.0001). The best cut-off value that differentiated control and stroke patients was calculated using a ROC curve (Fig. 1B). Table 2 indicates that a diagnosis of stroke may be established with 71.0% sensitivity and 90.3% specificity for a UFD1 cut-off of at 3.76 μg/L (AUC 0.90, 95% CI 0.82–0.98, *p* < 0.0001). This corresponded to 9 false negative and 3 false positive results out of 31 (OR 22.8, 95% CI 5.5–94.5; Fischer’s exact test, *p* < 0.0001). Interestingly, the control cohort included four patients suffering from various neurological conditions, including sleep apnea syndrome and meningioma (*n* = 1) and dementia (one with ischemic colitis and two with hip fracture) (*n* = 3). None of these patients showed positive results with the biomarker, strengthening its specificity. The plasma level of UFD1 was evaluated as a function of the time interval between blood collection and stroke onset. The ischemic stroke samples (established and transient) were divided into patients with interval below 3 h (*n* = 11) and above 3 h (*n* = 17). Figure 2 clearly shows a significant increase of the protein level within the first 3 h after stroke onset compared to the control sample (*p* < 0.0001, Kruskal–Wallis test with post hoc Dunn’s multiple comparisons).

**Figure 3:**
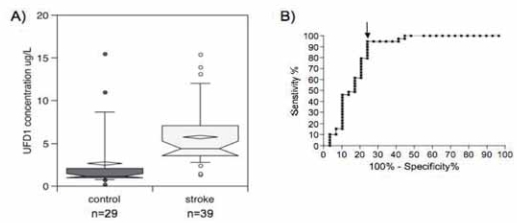
(A) UFD1 concentrations in stroke vs. control Spanish patients. Notched box and whisker charts show 10th, 25th, 50th, 75th and 90th percentiles and outliers. The diamond-shape indicates the mean. (B) UFD1 ROC curve. The arrow indicates the cut-off that gives the best sensitivity and specificity described in Table 2.

**Figure 4:**
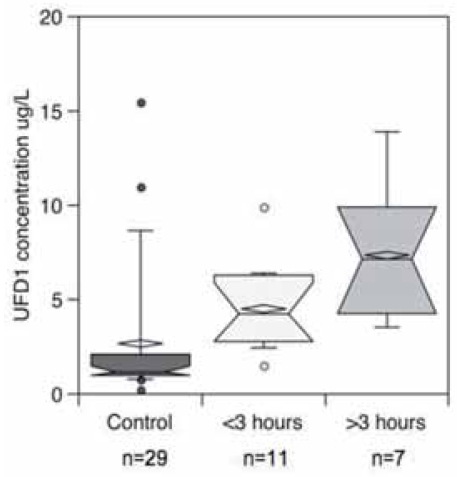
UFD1 concentrations as a function of time after the onset of the symptoms (Ischemic and TIA patients) for the Spanish population. Notched box and whisker charts show 10th, 25th, 50th, 75th and 90th percentiles and outliers. The diamond-shape indicates the mean.

A Spanish cohort comprising 29 control and 39 stroke patients and described in Table 1 confirmed these results. Stroke and control patients did not statistically differ with respect to age (*p* = 0.5934), Mann–Whitney test) and sex (*p* = 0.807, Fisher’s exact test). A significant increase of UFD1 was observed among stroke patients (*p* < 0.0001, Mann–Whitney test) (Fig. 3A). It was possible to diagnose stroke with 94.9% sensitivity and 75.9% specificity with a cut-off at 2.26 μg/L (AUC 0.84, 95% CI 0.73–0.95, *p* < 0.0001) (Fig. 3B, Table 2). This corresponded to 2 false negative out of 39 stroke patients and 7 false positive out of 29 control subjects (OR 58.1, 95%CI 11.1 305.2, Fischer’s exact test, *p* < 0.0001). No difference between stroke subtypes (hemorrhagic, ischemic and TIA) was observed (*p* > 0.05, Kruskal–Wallis test with post hoc Dunn’s multiple comparisons). As for the Swiss cohort, the ischemic stroke Spanish population (established and transient) was divided into patients with time onset of symptoms below 3 h (*n* = 11) and above 3 h (*n* = 7). About 10 hemorrhagic and 11 ischemic patients with unclear time onset of symptoms but below 24 h were removed from the study. Figure 4 clearly shows a significant increase of the protein within the first 3 h after stroke onset compared to the control population (*p* = 0.0004, Kruskal–Wallis test with post hoc Dunn’s multiple comparisons).

**Figure 5:**
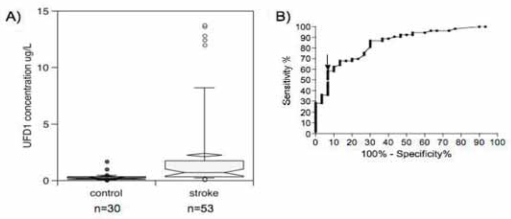
(A) UFD1 concentrations in stroke vs. control North-American patients. Notched box and whisker charts show 10th, 25th, 50th, 75th and 90th percentiles and outliers. The diamond-shape indicates the mean. (B) UFD1 ROC curve. The arrow indicates the cut-off that gives the best sensitivity and specificity described in Table 2.

UFD1 was also tested in a North-American sample described in Table 1. Box plots display a significant increase of UFD1 in stroke patients compared to controls (Fig. 5A). Using ROC curves (Fig. 5B), we determined that it was possible to diagnose stroke (hemorrhagic plus TIA plus ischemic) with 62.3% sensitivity and 90.0% specificity with a cut-off at 0.45 μg/L (Table 2) (AUC 0.85, 95% CI 0.76–0.93, *p* < 0.0001). This corresponded to 3 false positive out of 30 controls and 20 false negative out of 53 stroke patients (OR 10.7, 95% CI 3.3–35.3, Fischer’s exact test, *p* < 0.0001). Each subtype of stroke (hemorrhagic, TIA or ischemic) showed a significant increase compared to controls (*p* < 0.01, Kruskal–Wallis test, post hoc Dunn’s multiple comparisons, data not shown). No significant difference between stroke subtypes was observed.

Finally, we compared the performance of UFD1 with PARK7 and NDKA (two biomarkers previously validated in our laboratory) in the Swiss and North-American cohorts. Table 2 shows that UFD1 displays similar results as PARK7 and NDKA, in terms of sensitivity and specificity. In order to determine the best marker among the three tested, we compared areas under ROC curves (Table 2). Pairwise comparison of the six ROC curves (UFD1, PARK7 and NDKA in the Swiss and North-American studies) (Table 3) shows no significant differences (*p* > 0.05) except between UFD1 and PARK7 in the North-American studies (*p* = 0.005). However, this result was not confirmed within the Swiss cohort (*p* = 0.961).

## Discussion

The present small multi-centric study tested the hypothesis that UFD1 is increased in plasma and sera samples of stroke patients. This was demonstrated across three independent cohorts encompassing 90 control and 123 stroke patients with different genetic background (Swiss, Spanish and North-American). Assays were performed in two different places, Switzerland and the United States. Results in terms of sensitivity and specificity ranged from 62.3–94.9% for sensitivity and 75.9–90.3% for specificity. The comparison of UFD1 with PARK7 and NDKA within the Swiss and American cohorts shows that the three proteins display similar sensitivities and specificities. Comparison of the AUC did not allowed to determine the best marker among UFD1, PARK7 and NDKA within Swiss and North-American studies.

Using the Swiss and Spanish cohorts, we have demonstrated that it was possible to diagnose ischemic stroke patients early after a stroke event, i.e. within the first 3 h when a thrombolytic treatment can still be considered. In this context, it is hypothesized that UFD1 could be used as a follow-up marker for treated ischemic stroke patients. In addition, four controls with neurological disorders (dementia and meningioma) appeared negative for UFD1, suggesting specificity of the test regarding such disorders. Nevertheless, a large-scale study on stroke-mimic patients such as migraine, seizure or metabolic disorders should be performed. Moreover, 12 patients with high troponin I levels (acute myocardial infarction) did not showed significant elevated concentration of UFD1 compared to controls (data not shown). This result reinforces specificity of UFD1 as a diagnosis marker of stroke, compared to other vascular diseases.

The identification of UFD1 was based on its specific appearance in postmortem CSF presumably as a result of global brain ischemia and necrosis following death. We believe this model may partly reproduce mechanisms underlying the ischemic cascade of events leading to stroke lesions. The protein content of CSF has been examined in detail and many proteomic analyses of normal CSF (Finehout et al. 2004; Maccarrone et al. 2004), CSF from aging subjects (Zhang et al. 2005) or CSF from patients with neurological disorders such as multiple sclerosis (Hammack et al. 2004; Dumont et al. 2004), Frontotemporal dementias (Davidsson et al. 2002a; Folkesson Hansson et al. 2004) and Alzheimer’s disease (Davidsson et al. 2002b) have been recently published. However, the current knowledge of the protein content of CSF is likely to be largely incomplete and many more proteins will be identified in this biological fluid. It is therefore not surprising that Lescuyer and his co-workers only identified UFDI in postmortem CSF. This biomarker exhibits particular features, which makes it an appropriate candidate for reflecting aspects of ischemic brain injury. *UFD1L* gene encodes for the ubiquitin fusion degradation protein 1 (UFD1). UFD1 is highly expressed in adult heart, skeletal muscle and pancreas and to a lesser extend in placenta, lung, liver, kidney and brain. It was also found in fetal liver and kidney (Pizzuti et al. 1997). It is an essential component of the ubiquitin-dependent proteolytic pathway, which degrades ubiquitin fusion proteins. Deletions on chromosome 22q11.2 coding for UFD1L are associated with cardiac and craniofacial developmental defects that are characteristic of the DiGeorge syndrome (DGS), the velo-cardio-facial syndrome (VCFS) (Yamagishi et al. 1999; Driscoll et al. 1992) and the Opitz G/BBB syndrome (McDonald-McGinn et al. 1995) all known under the term of CATCH22 syndrome. A high prevalence of psychiatric disorders was observed in such patients and some groups have reported an involvement of *UFD1L* gene polymorphism in schizophrenia (De Luca et al. 2001; Bassett and Chow, 1999). The exact function of UFD1 in the pathogenesis of stroke is still unknown, but recently, it has been proposed that the ubiquitin-proteasome system contributes to neurodegeneration, in particular in Parkinsons’disease (Ross and Pickart, 2004) and to cerebral ischemic injury. Indeed, proteasome inhibition appears as a potential treatment in stroke by reducing neuronal and astro-cytic degeneration, cortical infarct volume, infarct neutrophil infiltration and NF-kB immunoreactivity with an extension of the neuroprotective effect at least 6 h after ischemic insult (see Wojcik and Di Napoli, 2004).

Based on the known distribution and function of UFD1, several hypotheses can be proposed regarding the mechanisms by which UFD1 may gain access to and be over-expressed in the blood of stroke patients. However, leakage through a disrupted blood–brain barrier (BBB) appears to be a likely, direct and nearly immediate route for UFD1 to appear in the blood.

In conclusion, UFD1 appears to be a reliable biomarker for the early diagnosis of ischemic stroke. As CT scan can exclude conditions such as tumor or hemorrhagic stroke, measurement of UFD1 plasma level could provide a powerful tool to rule-in ischemic stroke patients as a complement to clinical assessment and CT or MRI scan studies. Further investigations are currently underway to develop a 15-min lateral flow assay to evaluate prospectively UFD1 as a diagnostic marker of ischemic stroke and a follow-up biomarker in rtPA treated patients.

## Figures and Tables

**Table 1:** Demographic characteristics of Swiss, Spanish and North-American samples.

	Stroke	Control
Swiss cohort		
*n*	31	31
Age mean ± SD (min–max)	71.8 ± 15.6(25–92)	71.3 ± 16.0(28–91)
Female *n* (%)/Male *n* (%)	8(25.8)/23(74.2)	10(32.3)/21(67.8)
Time after onset of symptoms (h)	20.1 ± 30.1(0.5–120)	–
Mean ± SD (min–max)	3.9(2.1–48)	
Median (25–75 percentiles)		
Hemorrhagic *n* (%)	3(9.7)	–
TIA *n* (%)	6(19.3)	–
Ischemic *n* (%)	22(71.0)	–
Spanish cohort		
*n*	39	29
Age mean ± SD (min–max)	70.2 ± 12.1(44–95)	69.3 ± 9.5(54–87)
Female *n* (%)/Male *n* (%)	17(43.6)/22(56.4)	14(48.3)/15(51.7)
Time onset of symptoms (h)		
Mean ± SD (min–max)	10.03 ± 29.96 (0.5–150)	–
Median (25–75 percentiles)	3.05(1.92–7.27)	
Hemorrhagic *n* (%)	10(25.6)	–
Ischemic *n* (%)	29(74.4)	–
Cardioembolic PACI *n* (%)	5(12.8)	–
Cardioembolic TACI *n* (%)	4(10.4)	
Lacunar *n* (%)	5(12.8)	
Atherothrombotic PACI *n* (%)	5(12.8)	–
Atherothrombotic TACI *n* (%)	5(12.8)	–
TIA *n* (%)	5(12.8)	–
North-American cohort		
*n*	53	30
Hemorrhagic *n* (%)	6(11.3)	–
TIA *n* (%)	23(43.4)	–
Ischemic n (%)	24(45.3)	–

SD, standard deviation; PACI, partial anterior circulation infarct; TACI, total anterior circulation infarct; TIA, transient ischemic attack.

**Table 2:** Comparison of the performance of the three early diagnostic blood markers of stroke UFD1, PARK7 and NDKA.

	Ctrl *n*	Stroke *n*	AUC	Cut-off	SE%	SP%
	FP	FN	95% CI	(μg/L)	95% CI	95% CI
UFD1-Spanish	29 7	39 2	0.84(0.73–0.95)	2.26	94.9(82.–99.4%)	75.9(56.5–89.7%)
UFD1-Swiss	31 3	31 9	0.90(0.82–0.98)	3.76	71.0(52.0–85.8%)	90.3(74.2–98.0%)
UFD1-USA	30 3	53 20	0.85(0.76–0.93)	0.45	62.3(47.9–75.2%)	90.0(73.5–97.9%)
PARK7-Swiss	35 7	35 3	0.88 (0.80–0.97)	9.33	91.4(76.9–98.2%)	80.0(63.1–91.6%)
PARK7-USA	30 1	53 8	0.970.94–1.00)	1.55	84.9(72.4–93.2%)	96.7(82.8–99.9%)
NDKA-Swiss	31 3	31 3	0.94(0.86–1.00)	2	90.3(74.2–98.0%)	90.3(74.2–98.0%)
NDKA-USA	30 1	51 14	0.94(0.89–0.99)	2.55	72.6(58.3–84.1%)	96.7(82.8–99.9%)

Ctrl, control; FN, false negative; FP, false positive; SE, sensitivity; SP, specificity; CI, confidence interval.

**Table 3:** Pairwise comparison of ROC curves for the three biomarkers UFD1, PARK7 and NDKA for the Swiss and North-American cohorts.

	Difference between areas	Standard error 95% CI	Significance level p
UFD1_USA/UFD1_Swiss	0.064	0.069 −0.072 to 0.199	0.357
UFD1_USA/PARK7_USA	0.146	0.052 0.044 to 0.249	**0.005**
UFD1_USA/PARK7_Swiss	0.061	0.070 −0.076 to 0.198	0.383
UFD1_USA/NDKA_USA	0.096	0.055 −0.011 to 0.203	0.079
UFD1_USA/NDKA_Swiss	0.098	0.063 −0.026 to 0.222	0.122
UFD1_Swiss/PARK7_USA	0.082	0.045 −0.006 to 0.170	0.067
UFD1_Swiss/PARK7_Swiss	0.003	0.057 −0.108 to 0.114	0.961
UFD1_Swiss/NDKA_USA	0.032	0.056 −0.078 to 0.143	0.568
UFD1_Swiss/NDKA_Swiss	0.034	0.056 −0.076 to 0.144	0.541
PARK7_USA/PARK7_Swiss	0.085	0.049 −0.010 to 0.180	0.080
PARK7_USA/NDKA_USA	0.050	0.032 −0.013 to 0.114	0.123
PARK7_USA/NDKA_Swiss	0.048	0.038 −0.026 to 0.122	0.202
PARK7_Swiss/NDKA_USA	0.035	0.057 −0.077 to 0.147	0.539
PARK7_Swiss/NDKA_Swiss	0.037	0.046 −0.053 to 0.127	0.420
NDKA_USA/NDKA_Swiss	0.002	0.048 −0.093 to 0.097	0.966

## Abbreviations

AUC: area under curve
BBB: blood–brain barrier
CO: cut-off
CSF: cerebrospinal fluid
CT: computerized tomography
H-FABP: heart-fatty acid binding protein
MMP9: matrix metalloproteinase 9
MRI: magnetic resonance imaging
NDKA: nucleotide diphosphate kinase A
OR: odds ratio
RFU: relative fluorescence units
ROC: receiver operating characteristic
rtPA: recombinant tissue plasminogen activator
SE: sensitivity
SP: specificity
TIA: transient ischemic attack
UFD1: ubiquitin fusion degradation protein 1
